# The epigenetic–metabolic interplay in gliomagenesis

**DOI:** 10.1098/rsob.210350

**Published:** 2022-04-06

**Authors:** Bismi Phasaludeen, Bright Starling Emerald, Suraiya Anjum Ansari

**Affiliations:** ^1^ Department of Biochemistry and Molecular Biology, College of Medicine and Health Sciences, United Arab Emirates University, PO Box 17666, Al Ain, Abu Dhabi, United Arab Emirates; ^2^ Department of Anatomy, College of Medicine and Health Sciences, United Arab Emirates University, PO Box 17666, Al Ain, Abu Dhabi, United Arab Emirates; ^3^ Zayed Center for Health Sciences, United Arab Emirates University, Al Ain, Abu Dhabi, United Arab Emirates

**Keywords:** gliomagenesis, metabolism, epigenetics, tumourigenesis, organoids

## Abstract

Although tumourigenesis occurs due to genetic mutations, the role of epigenetic dysregulations in cancer is also well established. Epigenetic dysregulations in cancer may occur as a result of mutations in genes encoding histone/DNA-modifying enzymes and chromatin remodellers or mutations in histone protein itself. It is also true that misregulated gene expression without genetic mutations in these factors could also support tumour initiation and progression. Interestingly, metabolic rewiring has emerged as a hallmark of cancer due to gene mutations in specific metabolic enzymes or dietary/environmental factors. Recent studies report an intricate cross-talk between epigenetic and metabolic reprogramming in cancer. This review discusses the role of epigenetic and metabolic dysregulations and their cross-talk in tumourigenesis with a special focus on gliomagenesis. We also discuss the role of recently developed human embryonic stem cells/induced pluripotent stem cells-derived organoid models of gliomas and how these models are proving instrumental in uncovering human-specific cellular and molecular complexities of gliomagenesis.

## Graphical abstract

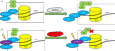

## 1. Introduction

Gliomas are brain tumours that mainly originate from glial cells, which are responsible for neuronal cell support and function [1]. Gliomas are the most common paediatric brain tumours and the main reason for death in children [2]. Brain tumours affect children at a rate of 5 per 100 000, and out of these, gliomas account for 75% of the cases [1]. Paediatric high-grade gliomas (pHGG) are the highly infiltrate brain tumours responsible for approximately 15–20% of all central nervous system (CNS) tumours in children and adolescents and have an incidence of approximately 0.85 per 100 000 children [3]. The typical treatment regimen includes surgery, chemotherapy and radiation, and the extent of surgical resection plays a crucial role in survival prediction. In adults, temozolomide treatment results in an increased survival period; however, no chemotherapeutic agents, including temozolomide, have shown any effect on the survival period in childhood HGGs [4]. For all age groups, initially HGGs respond to radiation; however, these highly invasive tumours' recurrence is inevitable.

The World Health Organization (WHO) classifies gliomas as astrocytomas, oligodendrogliomas, mixed oligoastrocytomas and other diffuse gliomas based on the glial tissue lineages, which closely resemble each other. Tumours are also graded from I to IV based on the malignancy of the tumour, with I–II as low-grade glioma. Grade III–IV astrocytomas and oligoastrocytomas are classified as high-grade glioma [5].

Most common among LGGs is the cerebellar pilocytic astrocytoma (PA) (WHO grade I), accounting for 85% of these tumours. PAs originate mainly in the cerebellum and the hypothalamic/chiasmatic region. Most of the remaining LGGs are diffuse astrocytomas (DAs) and pleomorphic xanthoastrocytomas, whereas diffuse oligodendroglioma or oligoastrocytoma is rare in children. The diffuse LGGs are invasive in nature, similar to pHGGs, anaplastic astrocytoma and glioblastoma [6]. In children, high-grade gliomas have the propensity to originate from the cerebral hemispheres, and almost 50% of these are diffuse intrinsic pontine glioma (DIPG) that originate from the ventral pons in the brainstem and occur most often in children. High-grade gliomas also occur in other midline structures, most commonly in the thalamus but also in the cerebellum and spinal cord, which are also mostly found in children than in adults [7]. Although the majority of gliomas are low-grade and are usually curable, up to 20% of paediatric gliomas are high-grade. High-grade gliomas are responsible for most cancer-related morbidity and mortality from infants to adolescents, with survival rates of only 10 to 15% [8]. According to WHO classification of nervous system tumours, high-grade diffuse gliomas (HGGs; grades III and IV) are distinguished from the low-grade diffuse gliomas with increased mitotic activity and nuclear pleiomorphism in grades III and IV glial neoplasms and with the addition of microvascular proliferation and necrosis in the grade IV tumours, glioblastoma [5].

For years, it was believed that the cause of cancer only relied on gene mutations. However, advances in understanding epigenetic biology led to clear evidence that aberrant epigenetic changes play a critical role in almost every step of tumour development and progression [9]. Recent understandings of the biology of pHGG revealed a strong connection between epigenetic dysregulation and pHGG development. Several studies have addressed the genetic and epigenetic mechanisms of pHGGs [10].

Dysregulation of epigenetic mechanisms such as chromatin remodelling and post-translational DNA/histone modifications may give rise to altered gene expression that leads to tumour formation [11]. The elucidation of these alterations is important for a better prognosis and if it is possible to revert these changes. This could also be valuable for novel drug development and for the improvement of current tumour-specific therapeutic strategies [10].

## 2. Epigenetic dysregulation in gliomagenesis

The nucleosome is the fundamental structural unit of chromatin in which a segment of eukaryotic DNA is wrapped 1.7 times by 147 base pairs around a nucleosome core comprised of a histone polyprotein octamer with two copies of histones H2A, H2B, H3 and H4 each [12]. Chromatin is critical for the regulation of transcription, replication, DNA repair and other features of genomic stability, including high fidelity chromosome segregation during cell division and maintenance of telomere integrity. Because of its central role in controlling critical cellular processes, an exquisite regulatory system called epigenetics of chromatin biology has evolved. This relies heavily on post-translational modifications (PTMs) of chromatin at both the DNA and histone levels [13,14].

A significant finding from recent tumour genome sequencing studies was the discovery that chromatin regulators, histone proteins, DNA modifications and nucleosome remodellers are frequently altered in malignancies [15]. Although altered chromatin states are recognized as a promising hallmark of cancer, genome sequencing analysis in 2012 contributed to the remarkable discovery that histones themselves could be mutated in cancer. Two groups have identified high-frequency somatic mutations in histone H3 in high-grade paediatric glioma (pHGG) [16,17]. These clustered missense mutations are always monoallelic and only affect one of the 16 genes encoding H3 in humans, including sites of well-established PTMs. These interesting aspects have attracted attention from oncology and chromatin biology researchers to resolve the mechanisms underlying these so-called oncohistones [18].

Several studies have shown that changes to the cellular epigenetic state could also result from the expression and incorporation of histone variants [19], further increasing the complexity of chromatin regulation. Except for H4, all histones are expressed in variant forms differing in primary amino acid sequence, with a structural dissimilarity. Interestingly, such variations were shown to have functional importance. For example, in humans, variants of H3 include H3.1, H3.2, H3.3 and CENPA [20]. CENPA is a histone of the H3 family that is deposited only at centromeres and structurally dissimilar to other H3 variants, whereas H3.3 differs from H3.1 and H3.2 in five amino acids. Several pieces of evidence suggest that H3.3 plays a distinct role in chromatin biology from canonical H3. Even though canonical H3 is expressed and incorporated into chromatin in a DNA replication-dependent manner, the expression, assembly and deposition of H3.3-containing nucleosomes are cell cycle independent [21,22]. The deposition of H3.3 into the chromatin is under the control of two different complexes. The deposition of it into transcriptionally active regions is modulated through the histone cell cycle regulator, while its deposition into heterochromatic regions, including that of the telomeric and pericentromeric regions, is through the DAXX (death domain-associated protein)/ATRX (thalassemia/mental retardation syndrome X-linked) complex [23].

Mutations in histone H3.3 are also identified to play a significant role in paediatric gliomagenesis. The recurrent mutations in the genes encoding histone variants H3.3, particularly *H3 Histone family member 3A* (*H3F3A*) and *H3.1* gene, *Histone cluster 1 H3 family member B* (*HIST1H3B*) are unique for paediatric gliomas [24]. In pHGGs, there are two mutations identified in the histone variant H3.3, G34 V/R and K27M. These missense mutations result in single amino acid substitutions that generate two mutants: K27M mutant, where the lysine of position 27 is replaced by methionine, and G34R or G34 V mutants where arginine or valine substitutes glycine at position 34 [16].

## 3. Histone modifications in pHGG

pHGGs of cerebral hemispheres has been shown to have H3.3G34R/V mutations where mutation occurs in only one copy of the H3F3A allele. Although the mutant residue G34R/V is not post-translationally modified itself, the mutant histone alters epigenetic regulation of the nearby lysine residue at position 36 (H3F3A-K36) [17]. The K36 position can be both methylated (K36me1/2/3) or acetylated (K36Ac). H3K36 trimethylation (me3) is a mark of transcriptional activation, which also plays a role in alternative splicing and DNA repair mechanisms. Mammalian cells contain around eight H3K36 methyltransferases, and among these, nuclear receptor-binding SET domain protein 1–3 and SET domain-containing 2 protein (SETD2) are considered major H3K36 methyltransferases. Only SETD2 can catalyse H3K36 trimethylation, and the rest of the enzymes catalyse H3K36 mono- and/or di-methylation, whereas lysine 36 acetylation is carried out by GCN5 [25].

**Figure 1.**
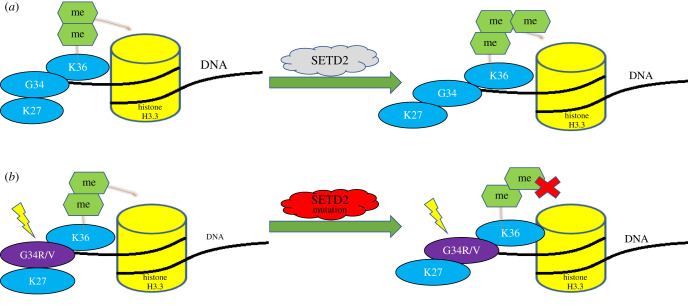
Epigenetic dysregulations related to high-grade paediatric glioma. (*a*) SETD2, an H3K36 trimethyltransferase that catalyses trimethylation of histone H3 and H3 variants. (*b*) Both SETD2 and H3.3G34R/V mutations affect H3K36 trimethylation, resulting in complete loss of H3K36 trimethylation without decreasing mono or di-methylation levels.

**Figure 2.**
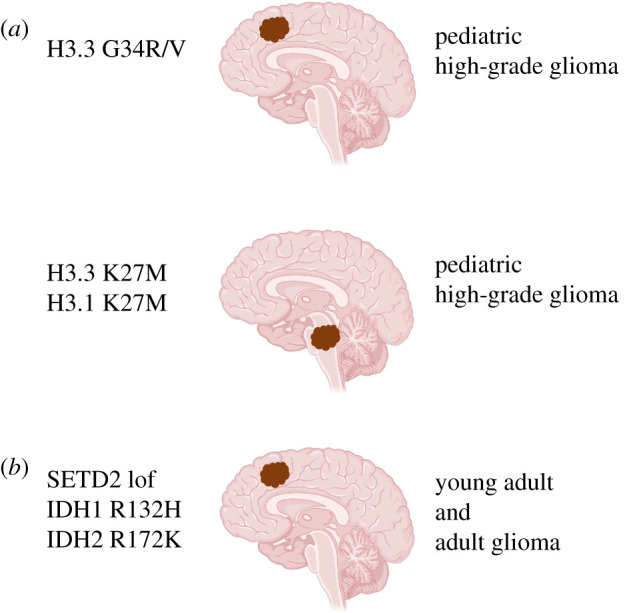
Chromatin regulator and/or metabolic enzyme driven paediatric and adult brain tumours. (*a*) paediatric high-grade gliomas (highlighted as red colour blobs) driven by H3.3 G34R/V or H3.3/H3.1 K27M mutations are located in the cerebral hemispheres and pons regions of the brain respectively. (*b*) Young adult and adult hemispheric gliomas driven by SETD2 loss-of-function (lof) mutations and IDH1 R132H or IDH2 R172 K (most frequent mutations of IDHs), respectively.

G34R/V mutations occur in histone H3.3 tail, and it shows a correlation with reduced H3K36 methylation due to the loss-of-function mutations in the SETD2 (figure 1). Mutations in SETD2 methyltransferase occurs exclusively in hemispheric HGGs and are primarily found in paediatric patients (15% of tumours) than adults (8% of tumours) [26]. These missense mutations were not observed in LGGs or midline structures (figure 2). Therefore, the anatomic location of these tumours is specific to particular mutations [27]. H3.3G34R/V mutant tumours also show a global DNA hypomethylation through the altered binding of H3K36 to its targets resulting in transcription activation, which in turn is mediated through the upregulation of MYCN oncogene [28,29]. Importantly, H3.3G34R/V mutant tumours were generally diagnosed during adolescence rather than in early childhood. Patients with G34R/V mutations in pHGG had a median age of 15 years and overall survival of 18 months, with a 2 yr survival of 27.3%. [24].The G34R/V mutation also defines a molecular subgroup of pHGG associated with loss-of-function mutations in the tumour suppressor protein 53 (TP53) and mutations in ATRX or DAXX [24,30].

ATRX and DAXX are components of a chromatin remodelling complex necessary for the incorporation of histone H3.3 at the pericentric heterochromatin of telomeres [7]. The loss-of-function of ATRX and, less frequently, DAXX affects telomeric stability and results in telomerase-independent maintenance through alternative lengthening of telomeres (ALTs) in H3.3G34R/V mutant tumour cells [24]. The ALT phenotype of G34R/V tightly fits with the loss of TP53/ATRX/DAXX; however, the role of TP53 in this process is not yet clear [7,16].

Mutations at lysine 27 (K27M) in H3.1 and H3.3, occurring in midline gliomas, are subjected to the position that normally undergoes PTMs. The histone N-methyltransferase, enhancer of zeste homologue 2 (EZH2), catalyses mono-, di- or trimethylation of K27 and induces gene silencing, whereas acetylation of K27 leads to activation of gene transcription and gene expression [31]. It is reported that K27M mutation mostly leads to reduction of H3K27me2/3 mark on nucleosomes resulting in transcriptional activation [32]. However, K27 mutation may also increase H3K27me3, leading to silencing of tumour suppressor gene expression [33]. H3.3K27M mutation is mostly found in the midline and pons and occurs in 63% of DIPG and in 59.7% of non-brainstem midline tumours (figure 2). At all anatomic locations, these mutations have a shorter survival period in patients (overall median 11 months; 2 yr overall survival 4.7%) compared to other mutations. H3.1K27M was highly specific to the pons (21.4%) representing the younger age group (median 5.0 yr) with a significantly longer overall survival (median 15 months) than H3.3K27M. Moreover, these mutations also co-occur with other mutations, as seen in H3.3K27M mutation, located in the pons, is shown to be associated with loss-of-function mutations of TP53 (60%) and gain-of-function mutations or amplification of platelet-derived growth factor receptor alpha (PDGFRA) (40%) [16,26], whereas mutation H3.1K27M in the pons region of the brain is shown to be associated with gain-of-function somatic mutations in the gene *activin A receptor type 1* (*ACVR1*) [34,35].

The histone N-methyltransferase, EZH2, is the family of polycomb repressive complex (PRC). The PRC 1 (PRC1) and PRC 2 (PRC2) compose the polycomb-group of proteins. PRC2 complex acts as a histone methyltransferase (HMT) through its subunits EZH1 and EZH2, and that trimethylates histone H3 on lysine 27 (H3K27me3), resulting in gene silencing [36]. A study by Lewis *et al.* [37] reported the inhibition of PRC2 activity due to the abnormal binding of K27M to EZH2. This aberrant recruitment of EZH2 causes a reduction of H3K27 methylation and leads to DNA hypomethylation [37,38], thus affecting gene activation and cell differentiation [39].

## 4. ATRX/DAXX mutations in pHGG

The ATRX protein is part of the SWItch/Sucrose NonFermentable family of chromatin remodelling proteins, and in combination with the transcription cofactor, DAXX (death domain-associated protein) maintains genomic stability through its deposition of the replication-independent histone variant H3.3 at telomeres and pericentromeric heterochromatin and other DNA repeat regions. DAXX was originally characterized as a Fas death receptor-binding protein that induced apoptosis via JNK pathway activation and thus was named as death domain-associated protein (DAXX) [40]. Within this ATRX/DAXX complex, DAXX carries out the histone chaperone activity, while ATRX plays the role of recruiting the complex to particular chromatin regions through interaction with histone modifications [41]. The loss of ATRX results in a deposition failure of H3.3 in the heterochromatic regions, which leads to genomic instability and DNA damage [23]. While the functions of ATRX is mainly associated with the deposition of H3.3 in the heterochromatin, recent studies have shown that it may also be involved with the deposition of H3.3 into actively transcribed euchromatin region and thus involved in its regulation. A recent study has shown the role of ATRX as an epigenetic regulator in glioma development [42], whereas the effects of ATRX mutations in ALTs, a non-telomerase-dependent telomere lengthening mechanism, are well established [43–46].

The absence of ATRX has also been strongly linked to DNA damage and replicative stress [47]. ATRX loss may occur by mutations, deletions or gene fusions and results in other molecular changes such as the ALT phenotype, PDGFRA amplification and TP53 (tumour protein P53) mutation [48].

In glioma, mostly all mutations in ATRX are inactivating, resulting in the loss of protein expression [49,50]. An interesting finding was that pHGGs harbouring G34 mutations in the *H3F3A* gene, and P53 mutations frequently have mutations in ATRX or DAXX, proteins that are part of the ATRX/DAXX complex.

However, K27M mutation in the *H3F3A* gene, which also usually co-occurs with TP53 mutations, coexist less frequently with ATRX mutations [16,51]. The role of ATRX/DAXX mutations in driving paediatric gliomas remains to be fully elucidated. However, one mechanism behind ATRX-driven tumours could be their effect on telomere length. Since ATRX inactivation triggers ALT, this mechanism allows the pHGG cells to extend their telomeres without the need for telomerase reverse transcriptase expression and constitutes a way to avoid death by telomere loss, allowing cancer cells for uncontrolled proliferation and cancer progression.

DAXX mutations are commonly found in pHGGs and paediatric DIPG and are usually mutually exclusive with ATRX mutations indicating that ATRX may be acting as an oncogene [52]. Moreover, DAXX mutations are not generally found in adult low-grade gliomas, indicating that DAXX-independent mechanisms of mutant ATRX may play a role in the phenotype of these tumour cells [23].

## 5. SETD2 mutations in pHGG

SETD2 (also known as HYPB or KMT3a) is a histone modification enzyme. *SETD2* gene encodes for the protein product, which is an enzyme, SETD2, that solely catalyses trimethylation of the lysine 36 residue on histone 3 (H3K36me3) in humans [53]. The functions of SETD2 are performed by several domains of this protein, namely the conserved SET domain, which mediates the methyltransferase activity, [54] whereas the Set2Rpd1 Interacting (SRI) domain associates SETD2 with the hyperphosphorylated carboxy-terminal domain of RNA polymerase II, and the WW domain precedes the SRI domain for mediating intramolecular interaction [55–57]. Even though biochemical evidence shows mono- and di-methylation activity of SETD2 in cells, it exclusively mediates trimethylation because SETD2 silencing leads to complete loss of H3K36 trimethylation without decreasing mono- or di-methylation levels in humans [58,59]. A decrease in H3K36me3 levels, in turn, alters gene regulation, increases spontaneous mutation frequency and causes chromosomal instability [60]. SETD2-inactivating mutations are also observed in different types of cancers, including in the CNS tumours [17,26] which are most predominantly found in paediatric and young adult high-grade gliomas located in the cerebral hemispheres and lead to 15%–28% and 8% of paediatric and adult high-grade gliomas respectively (figure 2) [26]. According to the WHO classification (2016), *SETD2* mutations are under the list of frequent mutations identified in paediatric high-grade diffuse astrocytic tumours that occur within the cerebral hemispheres. By contrast, recent studies show that SETD2 mutations are also found in low-grade gliomas, including DAs (WHO grade II) [61]. In high-grade gliomas, nonsense and frameshift mutations are specific to 5′ to the SET domain. By contrast, in low-grade astrocytic tumours, nonsense or frameshift mutations are mostly found 3′ to the SET domain [26], whereas missense mutations are found throughout SETD2 [61]. Moreover, SETD2 mutations may also work in conjunction with other mutations such as TP53 or mutations in growth factor pathways to promote tumourigenesis [62]. Although one of the most often mutated genes of high-grade gliomas is TP53, TP53 mutations were only seen in tumours with SETD2 missense mutations and not in those tumours with SETD2 nonsense or frameshift mutations. Frequently, the co-occurrence of SETD2 and TP53 mutations were seen in recurrent gliomas. These findings show that SETD2 mutations are not always synergized with TP53 mutations, and further studies are necessary [61] to understand their correlation. Other than TP53, the other most frequently observed genes showing mutations concurrent with SETD2 mutations within the high-grade glioma subset were EGFR and PTEN. Studies suggest that isocitrate dehydrogenase (IDH) mutations were present in a subset (18%) of diffuse gliomas with SETD2 changes, where all of the SETD2 mutations were missense rather than nonsense or frameshift mutations [26].

## 6. Metabolic dysregulations during tumourigenesis

Altered cell metabolism is a hallmark of cancers. Like all other cancers, gliomas rewire metabolism for increased cell proliferation, growth and survival functions, including macromolecule synthesis, ATP generation and antioxidant regeneration [63,64]. The tricarboxylic acid (TCA) cycle, also known as citric acid or the Krebs cycle, is the central pathway for cell metabolism that provides energetic intermediates and anabolic precursors via oxidization of acetyl-CoA into carbon dioxide [65]. Mutations in Krebs cycle enzymes are common in many cancers. In particular, IDH1 and IDH2 are present in over 80% of low-grade gliomas and a subset of adult glioblastomas (figure 2). However, mutations in IDH2 are much less common compared to that of IDH1 and are mutually exclusive.

IDHs are a group of enzymes playing an important role in the TCA cycle by catalysing the oxidative decarboxylation of isocitrate to *α*-ketoglutarate (*α*-KG), using nicotinamide adenine dinucleotide phosphate (NADP+) or nicotinamide adenine dinucleotide (NAD+) as a cofactor to generate NADPH or NADH during catalysis. Three different IDH isoforms (IDH1, IDH2 and IDH3) have been identified, with IDH1 performing its function both in the cytosol and peroxisomes, whereas IDH2 and IDH3 function only in the mitochondria [66,67]. Both IDH1 and IDH2 play important roles in many cellular functions, including glucose sensing [68], lipogenesis, glutamine catabolism and cellular defence against reactive oxygen species and radiation [69].

Mutation in IDH1 mostly affects Arg132 codon (R132), which is the binding site for isocitrate, and R132H is the most common alteration, comprising greater than 80% of all IDH1 mutations in gliomas [70]. Mutations in IDH2 solely affect Arg172 codon(R172) and Arg140 codon (R140), with the former structurally analogous to IDH1 R132 [71]. These loss-of-function mutations while losing its regular catalytic activity also gain the activity causing reduction of *α*-KG to produce the (R) enantiomer of 2-hydroxyglutarate, (R)-2HG (also known as D-2HG). These metaoboliets in turn accumulate in IDH1 or IDH2 mutated gliomas to millimolar concentrations [72]. Most IDH1 mutant tumours always retain one wild-type IDH1 allele, and disruption of the residual wild-type IDH1 allele decreases D-2HG production, which shows that IDH1 mutant-induced D-2HG production is probably dependent on wild-type IDH1 allele. This dependence has been proposed to be through substrate channelling or cooperative effects between the IDH1 wt/mut heterodimers [73]. However, the crystal structure of the IDH1 wt/mut heterodimer did not support substrate channelling as there was no physical association between the active sites of the wild-type and the mutant [74].

**Figure 3.**
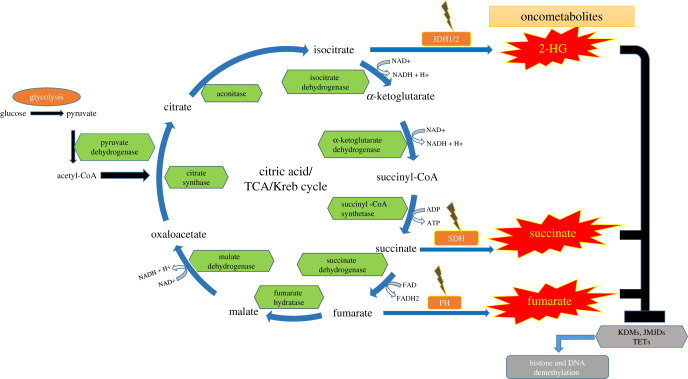
Metabolic dysregulation of TCA (Krebs) cycle due to mutations in the enzymes of this pathways generates oncometabolites that alter functions of epigenetic modification enzymes such as lysine demethylases (KDMs), Jumonji-C domain demethylases (JMJDs) and ten-eleven translocases (TETs), which in turn affect epigenome and gene expression pattern.

The IDH1 mutation leads to tumourigenesis through the control of the HIF-1*α* pathway, alteration of histone and DNA methylation, activation of glutaminolysis and increased sensitivity to glucose deprivation [75–77]. Mutant IDH gains tumourigenic properties due to its catalytic product D-2HG, which is considered an oncometabolite. Due to its structural similarity to alpha-ketoglutarate (*α*-KG), D-2HG inhibits DNA and histone demethylases, namely the ten-eleven translocase enzymes (TETs) [78] and lysine demethylases (KDMs), respectively [79], to block differentiation of non-transformed cells (figure 3). Mutant IDH1 promotes glioma growth in cells with PDGFA amplification and/or inactivation of CDKN2A, ATRX and PTEN [79].

Other Krebs cycle enzymes are also mutated in other types of tumours. Mutation or loss of fumarate hydratase (FH) can transform cells to become oncogenic and forms tumours leading to renal cysts and cancer [80]. Hypermethylation and reliance on pyruvate carboxylation are observed consistently in paragangliomas (PGLs) with succinate dehydrogenase (SDH) mutations [81]. Mutations in FH and SDH in these cancer cells cause an accumulation of fumarate and succinate, respectively which ooze out of the mitochondrial matrix and inhibit the prolyl hydroxylases (PHDs), leading to apoptotic resistance and hypoxia signalling even under oxygen-stable conditions (figure 3) [82]. Intriguingly, inhibition of PHDs has been shown to generate hypoxia-related signalling and pro-malignant behaviour in glioma cells [83].

### 6.1. Succinate dehydrogenase (SDH)

SDH is a key mitochondrial enzyme in the Krebs cycle and also functions as an electron pumping complex in the electron transport chain reaction in which reduced flavin adenine dinucleotide (FADH2) acts as a cofactor for its enzymatic activity [84,85]. SDH is comprised of four subunits (SDHA, SDHB, SDHC and SDHD), which are transcribed by four different genes, *SDHA, SDHB, SDHC* and *SDHD* and gets integrated into the inner membrane of the mitochondria. SDHA and SDHB are hydrophilic catalytic domains, while SDHC and SDHD are ubiquinone-binding and membrane-anchorage domains that are hydrophobic and anchor the catalytic subunits to the inner mitochondrial membrane. A functional unit, SDH complex assembly factor 2 (SDHAF2), which is encoded by the *SDHAF2* gene, has tumour-suppressive effects. SDH inactivation results in the accumulation of succinate and induces the stabilization of hypoxia-inducible factor (HIF)-*α* [86–88]. Succinate is a competitive inhibitor of HIF PHDs, which use *α*-KG to hydroxylate HIF-α, marking HIF-*α* proteins for polyubiquitination by the Von Hippel Lindau (VHL) complex [9]. Excessive succinate prevents the hydroxylation reaction, causing HIF-α to accumulate. Likewise, loss of VHL function prevents proper formation of the ubiquitination complex, resulting in HIF-α accumulation. Stabilized HIF induces pseudo hypoxic signalling and leads to angiogenesis, cellular proliferation and adhesion [82,86,89,90]. The accumulation of succinate may also be associated with alteration of epigenomic landscapes leading to oncogenesis through the inhibition of histone demethylation [91]. The inactivation of SDH can be caused by mutation of either of the *SDHA*, *SDHB*, *SDHC*, *SDHD* or *SDHAF2* (*SDHx* genes) [84,85,92].

Moreover, accumulation of succinate leads to the metabolic reprogramming of the ‘tumour microenvironment’, providing a favourable environment for tumour survival. Interestingly, mutations in different subunits of SDH complex lead to different types of tumours. For example, most mediastinal PGLs were related to SDHD gene mutations [93], whereas germline mutations of SDHB and SDHC play a minor role in sporadic head and neck PGL [94]. Genomic analysis of familial PGL/pheochromocytoma (PCC) has shown that while mutations of SDHD and SDHC are associated with PGL1 and PGL3, mutations in the large subunit genes SDHB, SDHA and SDHAF2 are associated with PGL4, PGL5 and PGL2 [95]. In renal cell carcinoma and papillary thyroid cancer, SDHB domain is mutated. Furthermore, mutations in SDHA, SDHB and SDHC have also been observed in gastrointestinal stromal tumours [96–98].

### 6.2. Fumarase or FH

FH is a homotetrameric enzyme of the TCA cycle found in the mitochondria that catalyses the hydration of fumarate to malate with three of its four chains combine to form the active site of the enzyme. Fumarase also acts as a tumour suppressor, and FH mutations in humans are commonly found in hereditary leiomyomatosis and renal cell cancer (HLRCC), a cancer syndrome characterized by a malignant form of renal cancer [99]. FH mutations were shown to be associated with cerebral cavernomas [100], neuroblastomas [101], Leydig cell tumours [102] and ovarian mucinous cystadenomas [103]. Intriguingly, some of the patients with FH mutations also develop PGL and PCC [104]. Like SDH mutations, FH mutations lead to loss of enzymatic function, and tumourigenic transformation also occurs due to the loss of heterozygosity of the wild-type allele [99,105]. FH inactivation results is the accumulation of fumarate, which has been shown to be an oncometabolite due to its role in the tumourigenesis process [106]. Like succinate, fumarate was shown to act as a competitive inhibitor of multiple *α*-KG-dependent dioxygenases, including histone and DNA demethylases, KDMs and TETs and HIF inhibitors, PHDs. By affecting these pathways, fumarate causes genome-wide epigenetic changes and HIF stabilization [107], both of which may contribute to tumour initiation, pseudo hypoxic signalling and lead to angiogenesis, dysregulation of cellular proliferation and adhesion [108] (figure 3).

## 7. Cross-talk between epigenetic and metabolic dysregulation in gliomagenesis

Metabolic alterations in cancer play an important role in gene expression regulation. Even though metabolic effects might have some impact at the genetic level, it shows a key role in the epigenome regulation [109–111]. Epigenetics, which mainly includes the modification of DNA and histones, is an inherent process that connects nutritional intake with cellular function. As a result, metabolic rewiring may cause cancer cells' epigenome machinery to transmit a mitogenic gene expression profile. At the same time, altered expression of genes involved in cellular metabolism is mediated by epigenetic dysregulation in cancer, suggesting a complex cross-talk between metabolome and epigenome during tumourigenesis [112–114]. This may depend on the availability of cofactors needed for epigenetic modifications, or the DNA/histone-modifying enzymes may be affected by metabolic rewiring. Also, the production of oncometabolites that act as the epigenetic modification enzyme's agonists and/or antagonists also affect the epigenome during tumourigenesis [115].

Mutations in the metabolic enzymes themselves could lead to carcinogenesis such as those discussed in the sections above. This includes IDH1/2 in gliomas [70] and acute myeloid leukaemia (AML) [79], SDH in PGLs [116] and FH in HLRCC [117] due to the production of oncometabolites, D-2-HG, succinate and fumarate. The common theme among these oncometabolites is their ability to competitively inhibit α-KG-dependent dioxygenases (*α*KGDs), which include TET (ten-eleven translocation) enzymes (TETs 1–3) that hydroxylate 5-methylcytosine (5-mC) to 5-hydroxymethylcytosine (5-hmC) which can promote DNA demethylation, the Jumonji family of histone KDMs and PHDs 1–3, which regulate HIF-1*α* and HIF-2*α* (figure 3) [118].

In AML, bioinformatics and functional studies indicate that a relevant target of D2-HG is TET2 which induces DNA hypermethylation and impaired differentiation in haematopoietic cells. These effects are caused partly through inhibition of TET2, a DNA demethylase enzyme that is also found to be mutated in some forms of leukaemia [79].

Similarly, in glioma, studies show that IDH mutations result in D-2HG accumulation, which triggers oncogenesis by altering the methylation pattern of DNA and histone [119]. Since the C-2 being a chiral carbon in 2HG, it has two enantiomers, L-2HG and D-2HG. For the clearance of 2HG, both enantiomers are removed by L2-hydroxyglutarate dehydrogenase (L2HGDH) [120] and D2-hydroxyglutarate dehydrogenase, and both these enzymes use FAD as an electron acceptor. They catalyse the oxidation of 2-HG and result in the production of *α*-KG and FADH2 [121] in the mitochondria. It has been reported that in clear cell renal cell carcinoma (ccRCC), enantiomer L-2HG is accumulated, resulting from the loss of L2HGDH copy number [122]. The transcriptomic and functional studies showed that increased L-2HG in ccRCC suppresses the dioxygenases, DNA demethylase TET and histone demethylase KDM6A, leading to DNA and histone hypermethylation. An increase in the expression of L2HGDH resulted in a decrease of L-2HG levels, and this helped in regaining the normal epigenetic state and the suppression of ccRCC. Therefore, L-2HG can also be considered as an oncometabolite [122,123]. Hence both enantiomers of 2-HG were found to inhibit *α*-KG-dependent dioxygenases, including TET2/and Jumonji-C histone demethylases by competitively inhibiting the *α*-KG binding site [78,124].

IDH mutant gliomas exhibit CpG island methylator phenotype [125], but the functional significance of this altered epigenetic condition is unknown. It was recently shown that in human IDH mutant gliomas, hypermethylation at cohesin and CCCTC-binding factor (CTCF)-binding sites compromised its binding with the methylation-sensitive CTCF insulator protein, resulting in an aberrant interaction of an enhancer with the receptor tyrosine kinase gene *PDGFRA*, which is a known glioma oncogene. Moreover, treating *IDH* mutant glioma spheres with a demethylating agent restored the function of an insulator and downregulated *PDGFRA* expression [126].

In an attempt to model low-grade astrocytoma, the introduction of IDH1R132H mutation and P53/ATRX knockdown in human neural stem cells resulted in the impaired neural stem cell differentiation and led to changes in DNA methylation. The differentiation blockage was caused by the downregulation of transcription factor SOX2, due to the dissociation of SOX2 promoter from its enhancer because of the disrupted chromatin looping caused by the reduced binding of the chromatin organizer insulator protein CTCF to its DNA motifs. Overall these findings underscore that mutated metabolic enzyme IDH promotes gliomagenesis by disrupting the crucial epigenetic mechanism, DNA methylation and allows aberrant regulatory interactions that induce oncogene expression [127].

Similarly, germline mutation of *FH* is reported in the type 2 tumour of HLRCC [80,128]. In these carcinomas, high levels of fumarate accumulation by loss of function of *FH* enzyme activity results in impaired function of TETs and affecting DNA demethylation within the genome [108]. Type1 and Type2 papillary renal cell carcinomas are two different types of renal cancer, where Type2 papillary renal cell carcinoma is caused by germline mutation of the *FH* [99]. These Type2 Tumours with CpG island methylator phenotype (CIMP) showed DNA hypermethylation of the *CDKN2A* promoter at loci that were unmethylated in the related normal tissue which shows a novel kidney-associated *CpG island methylator phenotype* (CIMP20) [15]. Interestingly, both germline and/or somatic mutation of *FH* (55.6%) led to downregulation of *FH* mRNA and upregulation of genes associated with cell cycle progression and response to hypoxia [129].

Moreover, loss of *FH* and fumarate accumulation affected TET-mediated demethylation and led to downregulation of the anti-metastatic miRNAs of the miR-200 family [130], leading to the activation of epithelial-to-mesenchymal transition (EMT) related transcription factors such as zinc finger E-box binding homeobox 1/2 (Zeb1 and Zeb2) and Snail homologue 2 (Snai2) and enhanced migration in renal cell carcinoma [131,132]. Proteome analyses of the mouse (Fh1^−/−^) and human (UOK262) FH-deficient cells identified Vimentin, a known EMT marker, overexpressed in these cells compared to FH-proficient cells. Further, gene expression profiling confirmed upregulation of other EMT-related genes in FH-deficient cells, whereas reintroduction of Fh1 (pFh1) in Fh1^−/−^ cells acquired an epithelial morphology compared to that of Fh1-deficient cells. Similarly, in UOK262 cells, Fh1 re-expression reverted the changes in Vimentin, Twist, Snai2, Zeb1 and Zeb2 expression. Moreover, miRNA profiling revealed that miR-200 family members were the most downregulated miRNAs in Fh1-deficient cells, whereas reintroduction of Fh1 in Fh1-deficient cells restored the expression levels of miR-200ba429 and suppressed Vimentin and rescues E-cadherin expression. To find out whether fumarate causes suppression of miR-200ba429 by inhibiting their Tets-mediated demethylation, silencing of both TET2 and TET3 in (Fh1fl/fl) Fh1-proficient cells resulted in decreased miRNAs and E-Cadherin expression. This suggests that changes in methylation levels of this region are, in part, caused *via* inhibition of Tets enzymatic activity. Moreover, 5-hmC, the oxidative product of 5-mC by Tets, was significantly decreased in Fh1-deficient cells. Incubation of Fh1-deficient cells with dimethyl *α*-KG (DM-*α*-KG), a cell-permeable derivative of *α*-KG, that reactivated *α*-KG-dependent dioxygenases restored miR-200a expression in Fh1-deficient cells [133]. On the other hand, treatment of Fh1fl/fl, (FH-proficient epithelial kidney cells) with monomethyl fumarate, a cell-permeable derivative of fumarate, showed significant phenotypical and epigenetic changes that resembled those of FH-deficient cells [130] (figure 3).

Interestingly, there is a bidirectional relationship between metabolic and epigenetic reprogramming in cancer [134]. Therefore, just as oncometabolites and metabolic dysregulations affect the epigenome, epigenetic impairments alter metabolism by influencing metabolic enzyme expression directly and/or by modifying signalling pathways that regulate cell metabolism [115]. For instance, mutations in the histone regulators/histone-modifying enzymes can cause metabolic dysregulation leading to tumourigenesis.

Deletion of SETD2 in renal cell carcinoma leads to metabolic reprogramming towards increased oxidative phosphorylation, enhanced TCA cycle metabolic enzyme activity and increased TCA cycle metabolite production in these cells. Furthermore, metabolic enzymatic activity assay showed a significantly elevated CS (citrate synthase) activity, the first rate-limiting TCA cycle enzyme in SETD2-deficient cells compared to control cells, although there was no apparent difference in CS at protein levels. GC-MS-based targeted metabolomics analysis showed significantly higher levels of aspartate, malate, succinate, fumarate and α-KG, and lower levels of lactate in SETD2 deficient cells [135]. Loss of SETD2 also affected gene networks related to glucose, mitochondrial and fatty acid metabolism, including upregulation of PGC1*α*, a master regulator for cellular energy homeostasis [136], which could have induced oxidative phosphorylation and TCA cycle gene expression, an increase in mitochondrial mass, cell size, and intercellular complexity SETD2-deficient cells [135] demonstrating the role of this histone-modifying enzyme in metabolic reprogramming in cancer.

Previous studies have also uncovered the role of lipid metabolism in gliomagenesis [137–139]. However, only recently, it was shown that ELOVL2, a key polyunsaturated fatty acid synthesis enzyme, is epigenetically upregulated in glioblasoma stem cells (GSCs) and is required for glioblastoma cell growth and tumour initiation. Interestingly, epigenetic upregulation of ELOVL2 is required for EGFR signalling which leads to GSC proliferation in glioblasoma [138]. The significance of this study also lies to the fact that it first identifies all superenhancers unique to glioblastoma cells and based on that identifies genes associated with it. This led to the identification of ELOVL2 as key gene of interest, suggesting that unique epigenetic programmes may be needed to modulte specific metabolic pathways for glioma cell survival and proliferation. This unique study again confirms the bidirectional relationship between epigenetic and metabolic programmes in cancer and further such studies are awited which will uncover the role of epigenetic alterations in driving tumour-specific metabolic programmes in glioma and other types of cancers.

## 8. Modelling gliomagenesis

In vertebrates, one of the earliest events in embryonic germ layer induction is the development of the CNS following the formation of the embryonic ectoderm [140].

The CNS is a complex tissue comprising a variety of cell types. Moreover, billions of neurones have to interact in an orderly manner to form functional neuronal networks. The CNS is formed during embryogenesis from being converted from a simple neural plate to a brain and spinal cord. To form many different types of neurones and glial cells in the adult CNS, embryonic cells have to proliferate and differentiate in a precisely controlled manner [141].

Rapid advancements have been made in the last few decades in understanding the molecular mechanisms underlying the initiation, proliferation and differentiation of the CNS at the cellular levels [141]. For a long time, mice, zebrafish and chickens have been used as model organisms to elucidate molecular mechanisms of embryonic development. However, these studies fall short in the ultimate effort to understand the mechanisms of human development to prevent and treat developmental abnormalities in humans. The lack of availability of human embryos and the inadequate amount of stage-specific and cell type-specific materials limit our understanding of the molecular interactions that underpin human development. Human embryonic stem cells (hESCs) may now be used to tackle these issues and seem to provide a valid model to understand complex signalling interactions occurring in human embryos [142].

**Figure 4.**
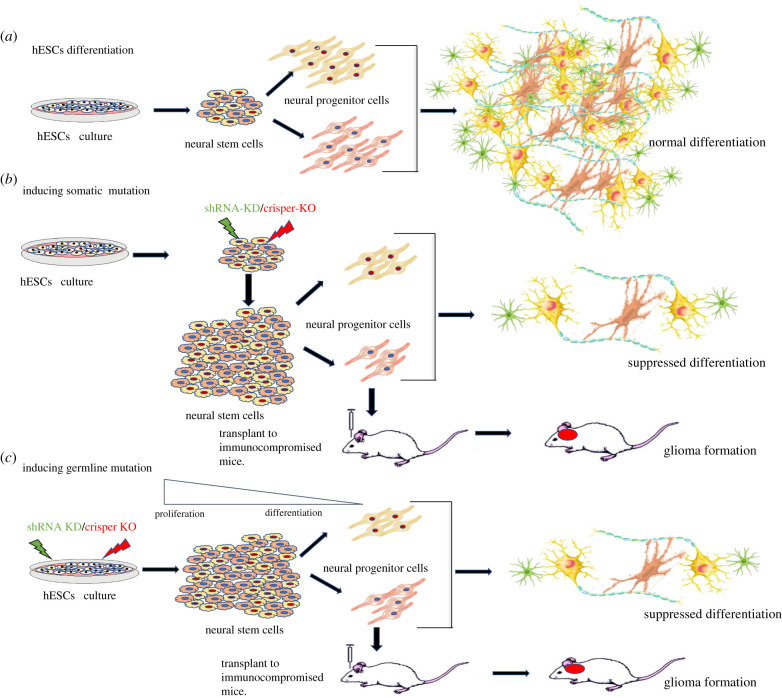
(*a*) Normal differentiation of hESCs towards different neural lineages in cell culture. (*b*) Mimicking somatic cancer driver mutations in neural stem cells or (*c*) germline mutations in hESCs using shRNAs mediated knockdown/CRISPER-Cas9 knockout could lead to increased proliferation of NSCs and defective cell differentiation resulting in cell transformation and tumourigenesis in cell culture models.

Human pluripotent stem cells, including hESCs and human-induced pluripotent stem cells (iPSCs), are the cells capable of expanding infinitely and differentiating into all human germ layers both *in vitro* and *in vivo* [143–145]. Differentiation of hESCs in culture follows the hierarchical set of signals that regulate embryonic development in the formation of the germ layers and specific cell types up to a certain extent. Thus, hESCs provide an alternative approach, as neural cells differentiated *in vitro* from hESCs show several characteristics similar to the developing embryonic brain [146]. Moreover, the ability of hESCs to differentiate into defined neural lineages, which include neurones, astrocytes and oligodendrocytes, is fundamental to study the sequence of events that take place throughout the early neurodevelopment [147,148]. Thus, hESCs can be considered as a suitable and valid system to study the significant roles of the signalling pathways involved in neural lineage commitment and model pathology of neurological diseases, including gliomas (figure 4) [142].

Several studies have identified gene mutations in malignant tumours, which are associated with pluripotency, suggesting a molecular relationship between these aggressive cancers and pluripotency. Thus, hESCs could be used as a model to investigate the early onset of neurodevelopmental diseases caused by genetic abnormalities [142]. hESCs also provide an appealing platform for functional analysis of neoplastic transformation in a genetically defined human background. Additionally, neural differentiation protocols allow the derivation of early neural stem cells that are often inaccessible; thus, tumour development can be well understood in terms of cell context (figure 4) [149]. For instance, in a study of paediatric glioma, hESCs were used to model tumours with H3.3K27M histone mutation. Using the dual Smad inhibition protocol, early neural progenitor cells (NPCs) from hESCs (H9, WA-09) were derived. hESCs-derived NPCs were then transformed using a combination of lentiviruses encoding H3.3WT or H3.3K27M plus PDGFRA and shRNA-P53. NPCs derived from H3.3K27M hESCs with P53 loss and PDGFRA activation resulted in neoplastic transformation. Transcriptome profiling of K27M transduced NPCs revealed these transformed precursors reset towards a developmentally more primitive stem cell state. This change occurs with major active H3K4me3 and repressive H3K27me3 histone marks at several master regulatory genes. Further, these neural derivatives of hESCs were used to screen for compounds that prevent tumour growth [149].

Another group showed that neural progenitors (NPs) derived from neoplastic human pluripotent parent cells possess brain tumour-initiating cell capacity (BTIC), similar to somatic tumours. Generation of neural tumours *in vivo* from neoplastic hESCs recapitulated early human paediatric brain tumours. NPs derived from transformed hESCs (t-hESCs) showed increased cell proliferation and could not mature towards the astrocytic lineage in both adherent cultures and neurospheres compared to progeny derived from normal hESCs (N-hESCs). Intracranial transplantation of NPs derived from N-t-hESCs and N-hESCs into mice led to neuroectoderm tumour formation exclusively from N-t-hESCs and showed classic medulloblastoma features, including retention of pluripotency [150].

In addition to hESCs, iPSCs are being intensively used for disease modelling [151,152] after Takahashi & Yamanaka [153] were able to reprogram adult somatic cells into pluripotent stem cells through the forced expression of Oct3/4, Sox2, c-Myc and Klf4 into mouse embryonic fibroblasts. iPSCs similar to human ESCs can give rise to cell types from any of the three germinal layers formed during embryogenesis (ectoderm, mesoderm and endoderm). As iPSCs are generated by genetic reprogramming of non-germ cells (somatic cells) and not from the embryonic tissues, these can bypass the ethical issues related to the use of embryos for hESC isolation. Therefore, the revolutionary discovery of iPSCs allows researchers to generate pluripotent stem cells without using embryos and provide a novel and powerful approach to ‘de-differentiate’ cells whose developmental fates had previously been considered to be predetermined. Hence, hiPSCs and/or their derivatives, with cancer-related genetic alterations, could represent a suitable strategy for developing human cancer models [154].

*In vitro* and *in vivo* hiPSC models for the study of human iNPC transformation to glioma tumour-initiating cells (GTIC)-like cells have been established recently. Genetic manipulation of P53 and receptor tyrosine kinase signalling led to the cancer stem cell-like features acquisition *in vitro*, such as enhanced self-renewal and migratory properties along with metabolic reprogramming. *In vivo* transplantation of transformed iNPCs resulted in the formation of highly aggressive tumours containing undifferentiated stem cells and differentiated derivatives. These results show hiPSCs as a suitable strategy for establishing tractable GTIC-like models recapitulating adult human glioma features [155].

Neurofibromatosis 1 (NF1) is a disease with clinical heterogeneity where affected individuals show a wide variety of pathologies, including brain tumours to developmental abnormalities. To model NF1 mutations' driven pathologies, a group of researchers engineered seven isogenic hiPSC lines, each with a different patient-specific *NF1* mutation [156]. The findings suggest that the germline NF1 gene mutation is one of the factors that cause clinical heterogeneity in patients with NF1. All heterozygous NF1 gene mutations showed an increase in CNS NPCs and astrocyte cell proliferation and RAS activity in two-dimensional cultures. Furthermore, cerebral organoids generated from the control and NF1-mutant hiPSC lines also showed an increase in RAS activity similar to the two-dimensional cultures in all NF1-mutant organoids. However, there were significant differences between NF1 mutations on two-dimensional cultured NPCs' dopamine (DA) levels and three-dimensional cultured NPCs' proliferation, apoptosis and neuronal differentiation in developing cerebral organoids. In NF1-mutant NPCs in two-dimensional cultures, DA levels were reduced by greater than 70% in three NF1-mutants but by less than 40% in the other four NF1 mutants relative to the control cell lines. Group 1 with three NF1 mutant organoids increased both proliferation and apoptosis during NPC differentiation, allowing neurogenesis to proceed normally. By contrast, group 2 with two NF1-mutant organoids had normal NPC proliferation, but reduced NPC apoptosis and very few immature neurones were present relative to the isogenic controls. In Group 2, the reduction in NPC death co-occurred with a delay in neurogenesis, suggesting that NPC subpopulations' survival creates a barrier to initiate neural differentiation in proper time. Thus, the use of the experimental human iPSC model revealed NF1 mutational defects in human NPCs and neurones which could not be observed before by using NF1 knockout animal models [156]. However, high degree of intra-tumour heterogeneity and the presence of different stem, progenitor and differentiated cells along the developmental hierarchy cause *in vitro* cell culture modelling of glioma more challenging [157,158]. Moreover, *in vitro* adherent two-dimensional monolayer cultures lack intrinsic heterogeneity, three-dimensional spatial organization and loss of interactions with components of the tumour extracellular matrix and the microenvironment. Also, these predict low treatment efficacy, as drugs that initially proved effective in the context of cultured cell lines might not show results in clinical applications [159].

Hence, more sophisticated model systems are needed to recapitulate complex cancer phenotypes, retain the tractability to perform detailed analysis and provide more accurate predictions of the therapeutic potential of novel drugs. Because of promising results in the other cancer fields [160–162], several laboratories have contributed to generating organoid models of glioblastoma, with three-dimensional structures in which different cell types self-organize appropriate cell-cell contacts to create a microenvironment [163]. The first three-dimensional model of GBM demonstrates glioblastoma stem cells were isolated from primary tumours, grown and expanded *in vitro* as two-dimensional adherent or three-dimensional neurosphere cultures with growth factors like epidermal growth factor (EGF) and fibroblast growth factor (FGF2). However, cells in neurospheres hardly mimicked *in vivo* GSC behaviour since they have lost their interaction with components of the extracellular matrix [158,164,165].

In 2016, the laboratory of Jeremy Rich developed an *in vitro* three-dimensional organoid model from human GBM cells and GBM biopsies. Fine pieces of GBM specimens were grown on Matrigel. These GBM organoids recapitulated the stem cell density gradient with respect to hypoxic levels present *in vivo*. Huge amounts of Sox2 + stem cells were expressed at the periphery of the organoid, while the core was characterized by lower Sox2 + cells with increased levels of hypoxia. However, this system was limited in relatively low to medium throughput capability and needed a long time to establish the cultures (one to two months) [166].

Recently, collaborative work between the University of Pennsylvania researchers led to developing a novel and faster protocol (one to two weeks) of three-dimensional GBM organoids. In this study, biopsies were cut into around 1 mm fragments and cultured on an orbital shaker in the absence of matrigel in serum-free conditions without EGF and FGF2. These glioblastoma organoids, called as GBOs, developed hypoxic gradients, histological features and cellular diversity of glioblastomas in culture. Moreover, these GBOs could be cryopreserved, recovered and continue growth upon thawing [167]. In 2018, the laboratories of Jürgen Knoblich and Inder Verma developed genetically engineered organoids to generate tumours. They screened for genetic alterations that could cause tumourigenesis and named the resulting tumour as neoplastic cerebral organoids: by overexpression of known oncogenes by a transposase-based system and/or deletion of tumour suppressor gene functions by CRIPSR Cas-9. Nucleofection was used to target organoid cells at the very early stages of the differentiation, and the cells containing genetic alterations were marked with GFP to track the cell growth and tumour transformation [168,169].

Recently, the laboratory of Howard Fine and others have developed a novel approach to study neoplasms by co-culturing patient-derived GSCs with three-dimensional brain organoids and named their model GLIoma cerebral organoids (GLICO) [170]. They co-cultured different GFP-marked GSC cell lines with completely grown cerebral brain organoids and showed that GSCs proliferate and integrate into the organoids over time. Moreover, co-cultured GSCs with a high degree of invasiveness, when transplanted in mice, were also more lethal [169]. Therefore, heterogeneity in growth and invasion in the GLICO model matches with certain intrinsic properties of specific patient-derived GSC lines [171].

Ogawa *et al*. [169] show that human cerebral organoids can be used as a model to study tumour formation in glioblastoma. They combined recent advanced human organoid and CRISPR genome engineering technologies to generate a genetically defined human glioblastoma (GBM). By introducing CRISPR/Cas9 and sgRNAs along with the activated oncogene HRasG12 V and disruption of the tumour suppressor, TP53, tumours were generated in human cerebral organoids. Transformed cells proliferated, showed invasive phenotypes within organoids and destroyed surrounding organoid structures, overwhelming the entire organoid. When transplanted into immunodeficient mice, tumour cells in the organoids exhibited uncontrolled invasive growth that led to the disease and death of the injected mice, which showed that organoid generated tumours exhibited characteristic features of tumourigenesis. Furthermore, expression profiles of these tumours were closer to mesenchymal subtype human glioblastoma. This further shows that human organoid-derived tumour cell lines or primary human-patient-derived glioblastoma cell lines can be transplanted into human cerebral organoids to establish invasive tumour-like structures. This technology allows the recreation of certain tumour-initiating genetic events and also to understand the natural history of tumour initiation of human gliomas, a process that usually is invisible in human cancer patients [169].

It is also worth mentioning the importance of recently developed *in vitro* blood–brain barrier (BBB) organoid model systems [172,173]. These organoids recapitulate *in vivo* characteristics of the BBB and can be used as a reliable and predictive tool to the study of gliomas and other CNS diseases and to investigate therapeutic potential of drugs. Bergmann *et al*. showed how BBB organoids can be grown within just 2–3 d by co-culturing endothelial cells, pericytes and astrocytes over a 24–48 h period under low adhesion conditions in a 1 : 1 : 1 ratio. Formed organoids were then incubated with different drugs of interest. Two different detection strategies, confocal fluorescence microscopy and mass spectrometry imaging were used to analyse the penetration of drugs into the organoids [172]. Utilization of such organoids could be effective not only in the trials of therapeutic agents but also to understand the molecular mechanisms of remodeling of the BBB, which is reported in several brain pathologies, including gliomas [174].

## 9. Concluding remarks

The recent identification of driver mutations in genes encoding histone proteins and chromatin regulators had established the role of epigenome dysregulations as central to the process of paediatric gliomagenesis as well as several other cancers [175,176]. Similarly, gene mutations in the enzymes of major metabolic pathways such as the TCA cycle have also been implicated in gliomas [177,178]. Not only do epigenetic and metabolic alterations drive the process of tumourigenesis, but there is extensive cross-talk among these two processes [179]. For instance, mutations in metabolic enzymes, IDH1 and IDH2, lead to the accumulation of D-2HG, termed as oncometabolite for its role in inhibiting the actions of α-KG-dependent dioxygenases, including DNA and histone demethylases. This results in defective DNA and histone methylations and supports the process of tumourigenesis [179]. Conversely, loss-of-function mutations in epigenetic modulators such as HMT, SETD2 results in metabolic dysregulations such as aerobic glycolysis and increased TCA cycle metabolite flux as observed in clear cell renal carcinoma [135], which are key features of metabolic rewiring in cancer cells. These results suggest a bidirectional relationship between metabolic and epigenetic perturbations in cancer. However, a lot remains to be understood about several other metabolic enzyme mutations, which could also result in tumour initiation and progression in terms of their cross-talk with the epigenome. For example, loss of function of several metabolic enzymes, as in the case of monogenic disorders termed as ‘inborn errors of metabolism’, is also found to be associated with carcinogenesis [180]. One such example is glucose-6-phosphatase-*α* (G6PC) deficiency which results in glycogen storage disease type Ia. A long-term complication of this metabolic disorder is hepatocellular adenoma/hepatocellular carcinoma (HCA/HCC) due to metabolic reprogramming of hepatocytes which includes increased glycogen storage, hepatic glycolysis, hexose monophosphate shunt (HMS) and lipid accumulation [181]. Some of the mechanisms of G6PC deficiency-mediated HCA/HCC includes hepatic autophagy impairment and activation of multiple tumour promoting pathways such as mTORC1 signalling, β-catenin and Yes-associated protein [182]. However, the role of epigenetic dysregulations due to G6PC deficiency-mediated HCA/HCC has not been studied to date. Importantly, driver mutations in metabolic enzymes associated with such cancers provide an excellent opportunity to study its effect on epigenome dysregulation due to dysregulation of one particular metabolic pathway or more extensive metabolic reprogramming. This would not only identify relationships between metabolome–epigenome dysregulations in such monogenic disorders but could also provide information for other cancers which also show similar metabolic rewiring during the process of tumourigenesis, such as gliomas.

Fortunately, combining recently developed cancer organoid models with the driver mutations in metabolic/epigenetic modulating enzymes has come with great opportunities to understand pathological mechanisms. Continued efforts in this area by modelling monogenic disorders in human ESCs/iPSCs derived organoid models will be instrumental in identifying novel mechanisms of tumourigenesis and possible therapeutic targets in future.

## Data Availability

This article has no additional data.
